# A Machine Learning Tool to Predict the Antibacterial Capacity of Nanoparticles

**DOI:** 10.3390/nano11071774

**Published:** 2021-07-07

**Authors:** Mahsa Mirzaei, Irini Furxhi, Finbarr Murphy, Martin Mullins

**Affiliations:** 1 Department of Accounting and Finance, Kemmy Business School, University of Limerick, V94PH93 Limerick, Ireland; mahsa.mirzaei@ul.ie (M.M.); finbarr.murphy@ul.ie (F.M.); martin.mullins@ul.ie (M.M.); 2 Transgero Limited, Cullinagh, Newcastle West, V42V384 Limerick, Ireland

**Keywords:** nanoparticles, antibacterial effect, antimicrobial capacity, biofilm, machine learning

## Abstract

The emergence and rapid spread of multidrug-resistant bacteria strains are a public health concern. This emergence is caused by the overuse and misuse of antibiotics leading to the evolution of antibiotic-resistant strains. Nanoparticles (NPs) are objects with all three external dimensions in the nanoscale that varies from 1 to 100 nm. Research on NPs with enhanced antimicrobial activity as alternatives to antibiotics has grown due to the increased incidence of nosocomial and community acquired infections caused by pathogens. Machine learning (ML) tools have been used in the field of nanoinformatics with promising results. As a consequence of evident achievements on a wide range of predictive tasks, ML techniques are attracting significant interest across a variety of stakeholders. In this article, we present an ML tool that successfully predicts the antibacterial capacity of NPs while the model’s validation demonstrates encouraging results (*R*^2^ = 0.78). The data were compiled after a literature review of 60 articles and consist of key physico-chemical (p-chem) properties and experimental conditions (exposure variables and bacterial clustering) from in vitro studies. Following data homogenization and pre-processing, we trained various regression algorithms and we validated them using diverse performance metrics. Finally, an important attribute evaluation, which ranks the attributes that are most important in predicting the outcome, was performed. The attribute importance revealed that NP core size, the exposure dose, and the species of bacterium are key variables in predicting the antibacterial effect of NPs. This tool assists various stakeholders and scientists in predicting the antibacterial effects of NPs based on their p-chem properties and diverse exposure settings. This concept also aids the safe-by-design paradigm by incorporating functionality tools.

## 1. Introduction

Antibiotic resistance is increasing to alarmingly high levels, the resistance mechanisms threatening our ability to treat common infectious diseases which leads to a global health risk [1]. Antibacterial agents are compounds that can be classified as either bactericidal, completely inhibiting and eradicating bacteria, or bacteriostatic, which inhibits bacterial growth [2]. However, several factors may influence this classification, including growth conditions, bacterial density or test duration [3]. More importantly, the effectiveness of most compounds depends on the type of bacteria (Gram-positive and Gram-negative bacteria) exposed to [2,4]. The majority of existing antibacterial agents are chemically modified natural compounds, e.g., β-lactamines (i.e., penicillin), cephalosporins or carbapenems; or purely natural products (i.e., aminoglycosides), and purely synthetic antibiotics, such as sulfonamides [2,5]. As a result of the recurrence of infections, the microorganisms develop resistance due to inherent genetic changes [6,7]. With the excessive use or misuse of antibacterial agents, the emergence of resistance to antibacterial drugs has become one of the most significant public health challenges [8,9].

While bacteria are normally found in nature in the form of individual cells, they may also develop multicellular structures called biofilms; densely packed groups of bacteria that contain one or more species of bacteria [10]. The biofilm provides mechanical stability and adhesion to a wide variety of surfaces both biotic and abiotic, including human tissues, surgical devices, implants, and industrial equipment [11,12]. Biofilms are a major issue in almost all industries, contaminating equipment and the surrounding environment, resulting in reduced quality of products and economic losses [13,14]. Bacterial biofilms contribute to microbial resistance and therefore play a significant role in therapeutic failure, resulting in chronic bacterial infections [15,16]. Considering the role of biofilm in antibiotic resistance, the elimination of bacteria needs multiple drugs with potential side effects in humans and environments, as a result of the need for high doses of common disinfectants and antibiotics there is the increase in toxicity, cost and duration of therapy; therefore, new treatments are necessary to eliminate bacteria.

Nanoparticles (NPs) are widely used due to their unique and size-dependent physical and chemical (p-chem) properties. They exhibit enhanced antimicrobial capacities [17], making them a suitable alternative to antibiotics. NPs have been studied for their capacity to inhibit microbial infections [18] and prevent bacterial colonization on various surface devices such as catheters [19] and prostheses [20] by eradicating biofilms [21,22]. Research into NPs is of great interest as they can be applied in various fields such as medicine, food industry, and manufacturing, while retaining their original unique functions [23,24,25,26]. Over the past few decades, the search for new antimicrobial substances has been central to many research areas, both in public and private research centers, for the reduction of nosocomial and foodborne infections [27,28,29].

Metal and metal oxide NPs (MO-NPs) are promising agents against a broad spectrum of microorganisms including drug-resistant strains [17,30,31]. The exact mechanisms of NP toxicity against different bacteria depends on surface modification, intrinsic properties, composition, and the bacterial species. The main mechanisms of the antibacterial effects of NPs are: (1) mechanical damage to the cell wall through electrostatic interaction; (2) oxidative stress by means of the generation of reactive oxygen species (ROS); and (3) disruption of cell and protein structures as a result of metal cation release [32]. Among MO-NPs, the most promising and widely studied ones are Fe_3_O_4_ and ZnO [33]. Fe_3_O_4_ NPs release Fe^2+^ ions, which cause the generation of ROS after a reaction with hydrogen peroxide (H_2_O_2_) and induces oxidative stress in the cell, as a result of which the bacteria cell dies [34,35]. ZnO NPs produce H_2_O_2_ and hydroxyl radicals (OH^−^), but not superoxide ($O_{2}^{-}$) and have weak mutagenic capability that induces frameshift mutation in bacterium [36,37,38]. Metal NPs such as AgNPs have an oligodynamic effect (the biocidal effects of metals) due to their large surface areas and have the ability to accumulate at the cell wall and bind with bacterial biomolecules [39] and penetrate the cells [40], generating ROS and free radicals, and act as modulators in the signal transduction pathways of microorganisms [41,42,43].

Antibacterial activities of NPs depend on two main factors: (i) p-chem properties, such as composition, surface modification and intrinsic properties, and (ii) the type of bacteria species [2,44,45,46]. For a better understanding of their properties and effects, a computational tool can assist in reducing the design space by predicting the characteristics of desired NPs before synthesis, which helps to decrease the experimental trial and error work. For example, tools have been employed to predict the three-dimensional structure of metallic nanoparticles [47,48] in order to determine the functional composition of the protein corona of NPs [49].

Artificial intelligence (AI) is a branch of computer science that has attracted much interest in many fields due to its problem-solving, decision-making and trend recognition capabilities. Machine learning (ML), a subset of AI, focuses on the ability of algorithms to learn from data while organizing the information they process. ML is a method of data analysis that automates model building while not requiring deterministic insights, bypassing in-depth comprehension and bridging input data directly to the outcome [50]. Moreover, these tools are fast and inexpensive, and they rely on information inputs rather than physical test materials. In addition, they can predict the impact of materials not yet synthesized, thereby contributing to safe-by-design approaches [51]. ML has been effectively employed for the prediction of toxicity profiles of NPs [52,53,54,55,56] and for the development of new antibiotics [57,58]. Furthermore, models for the prediction of the antimicrobial resistance for specific bacteria have been demonstrated [59,60,61]. For example, Khaledi, Weimann et al. [60] investigated the antimicrobial susceptibility of *Pseudomonas aeruginosa* predicted by genomic and transcriptomic markers. Yang, Niehaus et al. [62] employed algorithms for the identification of *Mycobacterium tuberculosis* resistance against several tuberculosis drugs. Her and Wu [59] demonstrated the prediction of antimicrobial susceptibility of *Escherichia coli* by using the pangenome-based ML approach.

To the best of our knowledge, there is no study that uses ML to predict the antibacterial effects of NPs. We propose an ML tool predicting the antibacterial activity of NPs against a vast range of Gram-positive and Gram-negative bacteria. This tool predicts the antibacterial effect by exploring p-chem properties, experimental exposure conditions and bacteria characteristics as inputs. We gathered in vitro experimental data from literature studies and structured them into a comprehensive dataset (Supplementary Material S1). The present approach allows the screening of NPs, predicting their capacity to eradicate bacteria, saving time and reducing costs by reducing the amount of trial and error in the lab. Such an approach would help scientists to prevent bacterial growth that can be harmful to human health, environments and industrial components that are subjected to biofilm formation and bacterial growth [63].

## 2. Materials and Methods

*Approach*

**Figure 1:**
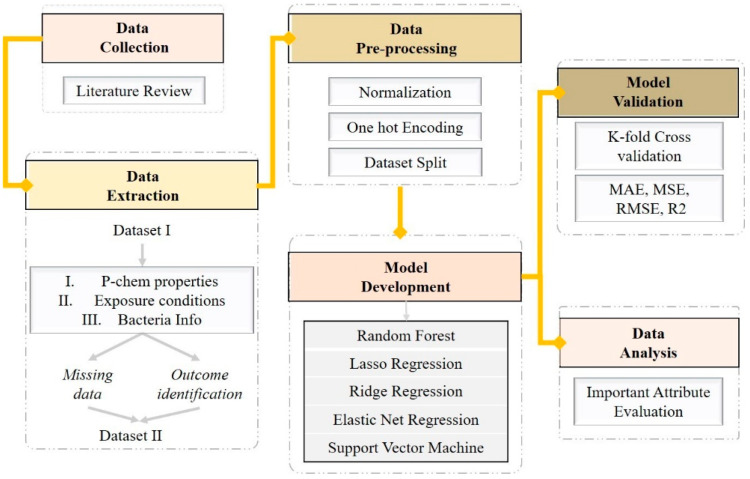
Model development workflow.

Figure 1 demonstrates the roadmap followed for the model implementation. Initially, studies related to the antibacterial effect of NPs are collected and data extraction is performed relating to p-chem properties of NPs, exposure conditions and information about the exposed bacteria. The original dataset was evaluated for completeness. Data pre-processing followed, including standardization [64], one hot encoding and one data split [65]. To find a model with good predictivity, we trained and validated several regression models. Finally, an analysis of attribute importance [66,67] was conducted to reveal the attributes that most influence the prediction of the results.

### 2.1. Data Collection

A literature search was carried out in January 2021 for studies that investigated the impact of NPs on the elimination or inhibition of bacteria and consequently the degradation of biofilm. The articles collected were published between 2010 and 2020 including different keywords, such as “antibacterial”, “antimicrobial”, “bactericidal”, “biofilm” and “anti-fungal” effects. We determined to evaluate silver (Ag) [68], iron oxide (IO) and zinc oxide (ZnO) NPs as they are widely used as bactericidal agents [17,69]. These NPs are promising candidates, since they demonstrate greater sustainability, reduced toxicity, greater stability and selectivity compared to organic NPs [70,71]. Their low toxicity against human cells [72,73], low cost [74], inhibition effect against a broad range of bacteria and inhibition of biofilm formation [75] makes them fitting for application as antibacterial agents in biomedical industries [76], food additives [77], fabric [75], and skincare products [78]. Studies using both chemical and green synthesis of NPs have been included. The green synthesis is a growing domain of bio-nanotechnology due to its low cost and non-toxic nature [69,79,80].

- Inclusion criteria for the studies include English language, original studies focusing on the antibacterial properties of NPs, published in the last decade, and in vitro studies.
- Exclusion criteria include reviews, case reports, studies with binary results, studies that demonstrated results only in figures.

A total of 85 papers were selected and 60 articles were deemed relevant to this study. We concentrated on in vitro studies due to the significant benefits they offer in agreement with the three R’s movement (replace, reduce and refine the animal experiments), reduced costs and allowing direct evaluation without the influence of pharmacokinetic variables [81].

### 2.2. Data Extraction

#### 2.2.1. Input Extraction

Each paper was reviewed with a focus on (i) the type of NPs (IONPs, AgNPs, ZnONPs) [2,82]; (ii) the nano-specific descriptors (core size, shape, zeta potential, surface area, etc.) [83]; and (iii) the study design experimental parameters (exposure conditions and bacteria characteristics) [84]. The above variables were acquired as input attributes for the prediction of the antibacterial efficiency of the investigated NPs.

#### 2.2.2. Outcome Extraction

For the evaluation of the antibacterial efficacy, studies reported different assays and techniques. Several outcomes based on antibacterial measurements were documented, such as bacteria viability, zone of inhibition (ZOI), minimum inhibitory concentration (MIC), minimum bactericidal concentration (MBC), colony-forming unit (CFU), optical density (OD), and inhibition in biofilm formation. Different metrics and expressions were shown in output values which stressed the fact that a standardized method and reporting of scientific data is required.

### 2.3. Data Pre-Processing

#### 2.3.1. Missing Values

In the initial raw dataset (Dataset I), missing data occurred in all the extracted attributes. Following the selection of the outcome, we created the final dataset (Dataset II). Our final data had few missing values among the inputs. As regression models cannot perform well with null data, we deleted the rows with missing values [85].

#### 2.3.2. One Hot Encoding

In regression models, categorical variables (nominal attributes) must be converted into integers (numerical dummy variables). There are several conversion methods [65]; we created dummy variables for each of the categorical columns and integrated the new columns into the main data frame. The value 0 or 1 was used to denote the absence or presence of the original attributes.

#### 2.3.3. Normalization

Data normalization was conducted on the numeric inputs to enhance model performance. Normalization is achieved by different techniques such as Z-score, min-max, mean and median absolute deviation scaling [86]. Normalization was done by applying a z-score that standardizes a feature to have zero mean unit variance [87].

(1) $$x^{\prime }i,n=\left(x\left(i,n\right)-\mu i\right)/\sigma \iota$$

where *x* represents the features in the dataset, *μ* the mean and *σ* the standard deviation of the *i*, *n* features.

#### 2.3.4. Data Split

For a supervised computational algorithm to predict outputs of an unknown target function, a training set is provided initially. We randomly split the data into two sets, one to train the model (training set) containing 70% of the dataset and the rest ones (30%) for testing the performance (test set) [88].

### 2.4. Regression Models

The regression technique constructs a model with the ability to predict new numeric values from the input variables. Regression modeling is the task of approximating a mapping function from inputs to a continuous output [89,90]. The ML algorithm maps functions from NP’s p-chem properties and experimental conditions to the inhibition of bacteria and enables the prediction of the antibacterial capacity of NPs. We used several supervised regression algorithms as potential candidates for developing our model to explore which model can provide the most accurate prediction. The Least Absolute Shrinkage and Selection Operator (LASSO) Regression, Ridge Regression (RR), Elastic Net Regression (ENR), Random Forest (RF) and Support Vector Machine (SVM) are examined in this study. Models were built in Python version 3.7.6, Scikit-learn version 0.24.1.

LASSO regression is a popular variable selection and shrinkage estimation method [91] which finds the variables and corresponding regression coefficients that lead to a model with higher accuracy. This is accomplished by imposing a restriction on model parameters, which then ‘shrinks’ the regression coefficients close to zero. Variables with a regression coefficient of zero after shrinkage are excluded from the model [92].

RR is a simple approach [93] that addresses the collinearity challenge arising in multiple linear regression [94,95,96]. When the covariates are super-collinear, two or multiple covariates are strongly related. When there is multicollinearity, least squares estimates are unbiased and their differences are larger, so they may be far from the real value. By adding a scale of bias to the estimates, RR decreases the standard errors. The RR model gives different importance weights to the features but does not drop unimportant features in comparison with LASSO [97].

ENR applies the penalties from both the LASSO and RR methods to regularize regression models [98]. ENR often outperforms LASSO, which is particularly useful when the number of predictors is much bigger than the number of observations [98]. This method aims to improve predictions by performing variable selection by forcing the coefficients of “non-significant” variables towards zero (shrinkage) [92].

RF comprises various decision trees that are trained independently on a random subset of data. RF can work with thousands of variables without deletion or reduction in accuracy, while preventing overfitting [99,100]. As a classifier, RF performs an implicit feature selection, using a small subset of “strong variables” for the classification, which is the reason for its superior performance on high dimensional data [101].

SVM learns by assigning labels to objects and is widely used in biological fields. It is used for both classification and regression challenges [102,103]. In SVM, each data item is plot as a point in an n-dimensional space with the value of each feature related to the value of a specific coordinate. It performs classification by finding the hyper-plane that differentiates the two classes.

### 2.5. Model Validation

The primary objective of ML is to generate an effective computational model with a high predictive capacity. Cross-validation is used to guarantee a stable assessment of the model performance and to avoid overfitting [104,105]. In cross-validation, the model is trained using parts of the training set by leaving one subset for later testing.

To produce an optimal model, a balance to avoid both underfitting and overfitting by adjusting hyperparameters is critical. In LASSO, RIDGE, and EN models, several statistical techniques were used to evaluate the model (data not shown) by using different sets of alpha values. To tune LASSO, RR and ENR models, we performed a “grid search” approach. The approach reduces the model’s complexity by keeping the most important features. The higher the alpha value, the more the regularization parameter influences the final model, hence decreasing the error due to variance (overfit). Alpha in regression models takes various values, however, when *α = 0*, the model gets same coefficients as in simple linear regression (no regularization).

The models were evaluated by mean absolute error (MAE), mean square error (MSE), root mean square error (RMSE) and Coefficient of Determination or R-squared (*R*^2^).

MAE is a popular metric, calculated as follows:

(2) MAE=1/n ∑i=1n|yi−yi^|

where *y_i_* is the *i*’th expected value in the dataset, yi^ is the *i*’th predicted value.

MSE is a standard and common error metric for regression model problems. The MSE is analyzed as the mean or average of the squared differences between predicted and expected target values in a dataset. It can be calculated by

(3) MSE=1/n ∑i=1n(yi−yi^)2

where *y_i_* is the *i*’th expected value in the dataset and yi^ is the *i*’th predicted value.

The RMSE of expected and predicted values can be calculated through the $\sqrt{\mathrm{MSE}}$. Although a good RMSE value is relative to a dataset, the smaller the value, the better the predictive model.

*R*^2^ is a statistical measure of fit that suggests how much variation of the output is supported by the inputs. *R*^2^ explains to what degree the variance of one variable describes the variance of the second variable. For instance, if *R*^2^ is 0.70, then approximately 70 percent of the observed variation can be explained by the model’s inputs, and the greater the R^2^ value, the better is the model.

### 2.6. Important Attribute Analysis

Attribute importance is a supervised event that distinguishes and ranks the attributes that are most important in predicting the outcome in a relative manner [106]. Attribute importance was derived through random forest optimization (built-in function). The analysis is based on the Gini importance, an all-nodes accumulating quantity that indicates how often a particular attribute was selected for a split, and how large its overall discriminative effect was in the regression [100]. Information values range from 0 to 1, with 1 representing maximum information gain.

## 3. Results

### 3.1. Data Pre-Processing

The primary dataset comprised 1176 rows and 18 columns (11 inputs, 7 outputs) extracted from 60 studies investigating the antibacterial properties of the NPs.

**Figure 2:**
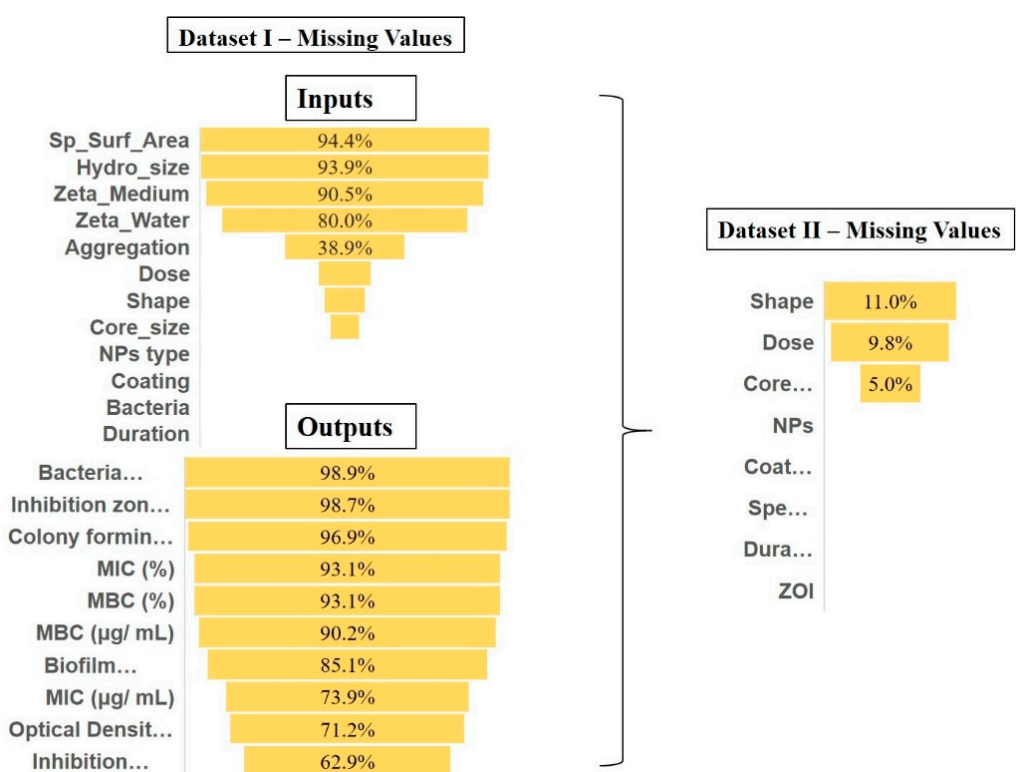
Dataset I. Missing values (percentages) of input and output parameters (left). Dataset II missing values of inputs and one outcome, ZOI (right).

Input selection and transformation: The input data consisted of specific surface area (m^2^/g), hydrodynamic size (nm), zeta potential (measured in water and medium) (mV), core size (nm), exposure dose (μg/mL), and duration (h) reported in numeric values. Variables with nominal values included shape, type of NPs, coating, bacterium and aggregation as summarized in Table 1. As can be seen from Figure 2 (left), specific surface area, hydrodynamic size and zeta potential had approximately more than 90% missing values and were therefore excluded. The aggregation potential had 39% missing data and therefore was discharged. The different types of NPs, coating, duration, and bacteria had no missing values. The dose, shape, and core size had 17.3%, 13.5%, and 9.6% missing values, respectively.

**Figure 3:**
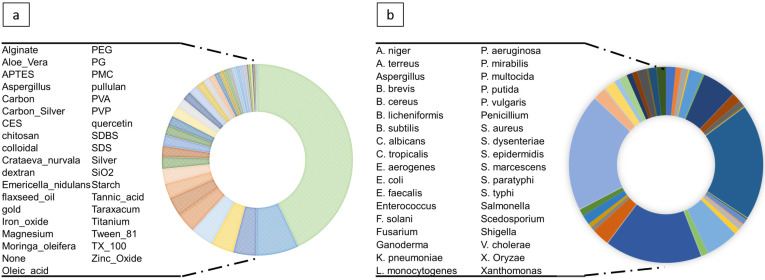
Coating information of NP, the coating variables are all 57% and the un-coated is 43% (**a**). Species of several investigated foodborne and environmental bacterium (**b**).

The coating and bacteria variables were very dispersed (as illustrated in Figure 3). To prevent model overfitting, we transformed coating into coated and uncoated (binary format). The studies were conducted on several strains of bacteria for which we collected the class, family, and species information. To avoid overloading the model we only kept species as a subcategory of class and family, grasping general (Gram-positive or negative bacteria) and specific information. In the final dataset (Dataset II), we included shape with 11%, dose with 9.8% and core-size with 5% missing values. The other variables had no missing values.

Outcome Selection: We gathered all the evaluations used to determine the antibacterial efficacy of the NPs. Each of the outcomes has different unit metrics. For example, the bacteria viability/growth is expressed in the number of bacteria cells and cell viability is reported as a percentage of live bacteria. ZOI is given in mm, representing the diameter of the area of media where bacteria are unable to grow [107]. MIC is extracted in μg/mL as the minimum concentration of NPs that inhibit the growth of bacterium [107]. MBC is expressed in μg/mL as the lowest concentration of antibacterial agent required to kill a bacterium [108]. OD measurements represent growth analysis by measuring the optical density at different settings of 580, 600 and 572 nm. The biofilm formation is reported in a percentage and change in biofilm growth is reported in colony-formed unit (CFU) [109].

Due to the high occurrence of missing data and diversity in the outcomes (as shown in Figure 2), we chose the outcome with the least missing value, the inhibition zone measurement (ZOI) in mm, with 60% missing values. Hence, the rest of the outcomes were dismissed in Dataset II. ZOI testing, also known as the disk diffusion method (DDM), is a fast and inexpensive assay compared to the other laboratory tests [110,111]. The diameter of the ZOI illustrates the antimicrobial activity present in the sample or product—a larger zone of inhibition means that the antimicrobial is more potent. In summary, the final dataset of 436 rows consists of seven inputs (shape, dose, size, coating, type of NPs, bacteria species, and duration as inputs) and one output (ZOI) derived from 60 studies.

### 3.2. Validation of Models and Attribute Analysis

**Figure 4:**
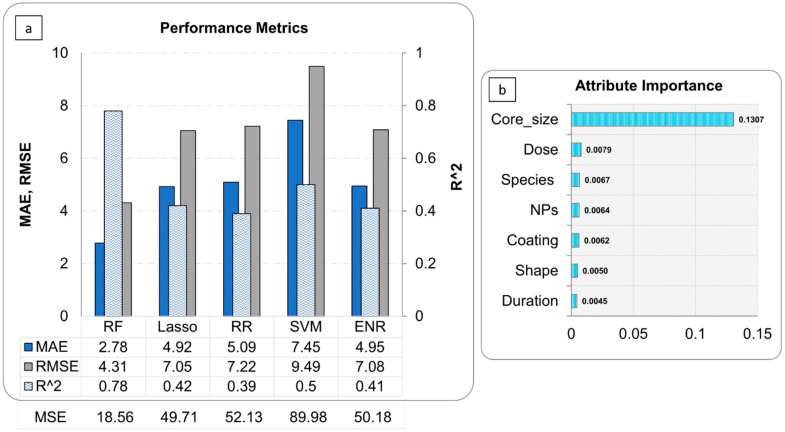
Performance metrics (**a**) and Random Forest Attribute Importance analysis results (**b**).

Following data homogenization and pre-processing, we trained various regression models. Model performance results are presented in Figure 4a. The results suggest that the RF model exhibits the lowest error and the highest *R*^2^ score compared to the other algorithms employed (LASSO, RR, ENR, SVM), with *R*^2^, RMSE, MAE and MSE of 0.78, 4.30, 2.78 and 18.56 values, respectively. The outcome of attribute importance analysis is shown in Figure 4b. Core-size is the most important attribute that determines the efficacy of the antibacterial effect of NPs. The dose, species, and type of NPs are identified as comparatively important, followed by coating, shape, and duration. Further data analysis such as correlation and batch effects are presented in Supplementary Materials (S2).

## 4. Discussion

In the present study, we implemented an ML tool from data collection to model validation, to predict the bactericidal effects induced by NPs in in-vitro systems. The model is consistent with the OECD principles [112] addressing the selection of (i) a defined endpoint (zone of inhibition as a metric to evaluate the susceptibility of the bacteria to NPs); (ii) an explicit algorithm (RF, https://github.com/mahsa-mirzaei/RFR_ABA.git, accessed on 6 July 2021); (iii) a well-defined domain of applicability (data ranges and nominal categories are provided, Table 1); and (iv) appropriate measures of goodness-of-fit; robustness and predictability.

Recent studies have demonstrated that p-chem properties such as core-size [113], shape [114], surface area [115], zeta potential [116], aggregation [117], and hydrodynamic size [115] play an important role in determining the antibacterial activity of NPs [116,118]. Exposure conditions such as dose play an important role as well [119]. For the above reasons, we gathered information regarding the p-chem properties of NPs and exposure conditions. However, the appearance of missing data among our input was significant. For instance, specific surface area, hydrodynamic size, and zeta potential were absent in more than 90% of our data, aggregation information was 39% missing. Although this is essential information, regression tools do not perform well with missing data. P-chem properties are important factors and should be mentioned in future studies. Another point is the appearance of multiple diverse coatings found in the literature. In our dataset we found thirty-seven coatings (less than 4% each and 43% uncoated). For the moment, the dataset is not sufficiently large to distinguish the influence and variance of each coating to the antibacterial capacity since the models overfit (reduction in predictive power).

In the studies reviewed, different methods to determine the NP size and morphology, such as Transmission Electron Microscopy (TEM), Scanning Electron Microscopy (SEM), Differential Mobility Analyzer (DMA), Dynamic Light Scattering (DLS), X-ray scattering, and UV–vis absorption spectrum, were reported. The presence of coating was assessed using electron microscopy combined with X-ray (energy-dispersive X-ray spectroscopy, EDX) or X-ray fluorescence spectroscopy (XRF) measurements. The specific surface area was measured by N2-BET, Ultra X-ray photoelectron spectroscopy technique, and NMR. The zeta potential was measured in different media (water and culture media) by electrophoretic mobility using Henry’s Equation and the Schmolukowski approximation. The above summary signifies the need for a standardized characterization workflow to obtain homogenized data across different studies.

The reviewed studies captured the antibacterial effects of NPs by using different protocols. To create a consistent dataset, the experimental data should be generated by a single protocol, but this is impractical. For the outcome we focused on one antibacterial evaluation method to obtain uniform data, which represented only 40% of the gathered data. Subsequently, it demonstrates the need for harmonized and rigorous methods used to evaluate the antibacterial activity of NPs to accomplish reproducibility as well as reliability.

RF has been effectively utilized in various domains, e.g., in microbiology and genetics, and has become a major data analysis tool due to its superior performance on high dimensional data [101,120,121]. Our results show that RF predicts the antibacterial effect more acutely compared to other models due to the determination of the non-linear relationship between input and output variables. The second-best model was LASSO. The key challenge with LASSO is correlated variables, in that it retains one variable and sets the other to zero. This will lead to some loss of information resulting in lower accuracy. In order to evaluate the reliability and performance of the resulting models, we assessed the goodness-of-fit, robustness and predictivity by MSE, MAE, RMSE, and *R*^2^ statical methods. The closer the value of R^2^ (measures of goodness-of-fit) to 1, the better the model is fitted [122]; smaller values of MSE, RMSE, and MAE verified model performance [123,124].

Several techniques exist for pre-processing data to make them suitable for use in computational tools, such as normalization, one hot encoding, and feature selection handling of missing values. One technique worth noting is the description of molecular structures [125]. The most common methods to codify structures are (i) the chemical graph; (ii) the notations as Simplified Molecular Input-Line Entry System (SMILES); and iii) the de-facto standard chemical formats. Experimental and exposure conditions are vital variables in the representation of antibacterial capacity since the same type of NPs may exhibit diverse effects in different experimental conditions. This makes the development of classic QSAR difficult [126]. Toropova et al. [127] suggested a quasi-SMILES approach to represent molecular structures, p-chem properties, and experimental conditions (eclectic data) with NPs [126,128].

There was no ML study to compare with as to which parameters are the most important to predict the antibacterial activity of NPs on the model performance. We based our evaluation on the attributes that are investigated by researchers in the lab [129]. For example, according to the literature, various parameters such as core size, dose, shape, and special surface area of the NPs affect the antibacterial activity of NPs [118,130].

- Size: The smaller the particle size, the higher the antibacterial activity [118]. This can be explained by the fact that NPs can easily cross the bacteria membrane and reach the nuclear; secondly, because of a larger surface/volume ratio [131,132,133,134].
- Species: Type of bacteria is important in determining the antibacterial activity of the NPs [135,136,137]. Depending on their cell wall composition, bacteria are divided into two groups: Gram-negative and Gram-positive [138]. Various NPs with different surface charges can act distinctly depending on what the differentiation is in the bacteria cell wall composition [132,135].
- Dose: A dose-dependent reduction of bacterial growth and biofilm biomass is observed following exposure to metal and metal oxide NPs [139,140]. Remarkably, our findings, according to the attribute important analysis, confirm that the core size, dose, and bacteria species are the most important attributes affecting the prediction of the antibacterial activity of NPs.

More systematic data are needed to enable building models accounting accurately for the all the possible in vitro determinant combinations. Without agreement on standard characterization workflow of NPs or defined key properties that define their efficacy and a lack of reference bacteria and defined assays, there is still a long way to go to unravel systematically the antibacterial properties of NPs. In addition to precise protocols and standardization of methods, there should be further harmonized outlines in how to report p-chem properties of NPs or experimental conditions and to make those measurements more comparable to improve the reporting data. The absence of comprehensive metadata description for related bioassays may have an impact on the clarity and comprehension and therefore the quality of the results [141]. Several different initiatives are currently working on defining frameworks, methods, and criteria for evaluating the quality of the reported data based on the FAIR (Findable, Accessible, Interoperable, Reusable) data principles [142].

## 5. Conclusions

Antibiotic resistance of bacteria has become one of the major concerns in human healthcare. NPs represent a valuable and innovative technology to build alternatives to antibiotics. In this study, we investigate the performance of various ML tools to predict the effects of NPs as an antibacterial agent on vast groups of Gram-positive and Gram-negative bacteria, with RF being the best model. This study is a first step and the first tool to assist researchers towards screening NPs with potentially high antibacterial effects and could help fine-tune their properties. With this interdisciplinary approach we combine knowledge of the underlying science with computer science tools. This is a valuable activity as it allows those working in the laboratories to leverage development in the AI space and thus improve timeliness, reducing the number of experiments performed and the costs involved. Due to the inconsistency of reporting NP p-chem properties in antibacterial studies, the resulting dataset has large data gaps. We highlight the need for standardized measurements to evaluate the properties of NPs, allowing more consistent metadata. The majority of data in the literature revolves around the toxicity of NPs. This paper stresses the need for more data, raising awareness to the scientific community of the lack of comprehensive datasets regarding the antimicrobial capacity of NPs.

## Figures and Tables

**Table 1:** The primary and final Input variables in Dataset I and Dataset II.

			DATASET I	Data Transformation	DATASET II
Category	Variables	Type	Min Max or Labels		
P-chem properties	Sp_Surf_Area	Numeric	1.2–96 (m^2^/g), NA	Eliminated due to high NA	−
	Hydro_size		11.5–993 nm, NA		−
	Zeta_Medium		−40–90 (mV), NA		−
	Zeta_Water		−40–80 (mV), NA		−
	Core_size		2–1000 (nm), NA	Selected	4–546 (nm), NA
	Aggregation	Nominal	Yes, None, NA	Eliminated due to high NA	−
	Shape		Spherical, Hexagonal, Rod, Spindle, Disc, Cubic, NA	Selected	Spherical, Hexagonal, Rod, Cubic, NA
	NPs type		AgNPs, Fe_3_O_4_, ZnO		AgNPs, Fe_3_O_4_, ZnO
	Coating		Iron Oxide, dextran, pullulan, Taraxacum officinale, Aspergillus, Emericella nidulans, Tannic acid, quercetin, TXT_100, SDBD, SDS, Tween 81, PEG, PVP, Crataeva nurvala, PMC, PG, Moringa oleifere, Oleic acid, Zinc oxide, Gold, Chitosan, APTES, Flaxseed oil, silver, CES, alginate, PVA, Carbon, Alow vera, Titanium, SiO_2_, starch, Magnesium	Simplified: Transformed into Binary	Coated, Uncoated
Exposure	Dose	Numeric	0.01–10.000 (μg/mL), NA	Selected	0.01–10.000 (μg/mL), Na
	Duration		17–1440 (h)		17–1440 (h)
In vitro Info.	Bacteria	Nominal	*Acetomicrobium faecale*, *Acidaminococcus fermentans*, *Actinomyces denticolens*, *Aspergilus* (*niger*, *terreus strain*), *Bacillus brevis*, *Bacillus cereus*, *Bacillus subtilis*, *Bacteroides* (*eggerthii*, *stercoris*, *thetaiotaomicron*, *uniformis*, *vulgatus*, *xylanolyticus*), *Bifidobacterium* (*adolescentis*, *bifidum*, *longum*, *suis*, *thermophilum*), *Campylobacter jejuni*, *Candida* (*albicans*, *parapsilosis*, *tropicalis*), *Citrobacter freundii*, *Clostridium* (*butyicum*, *cellulovorans*, *coccoides*, *histolyticum*, *leptum*, *perfringens*, *thermocellum*), *Corynebacterium glutamicum*, *Enterobacter* (*aerogenes*, *cloacae*), *Enterococcus* (*cecorum*, *durans*, *faecalis*, *faecium*, *hirae*), *Escherichia coli*, *Eubacterium eligens*, *Fusarium solani*, *Ganoderma*, *Klebsiella* (*aerogenes*, *oxytoca*, *pneumoniae*), *Lactobacillus* (*acidophilus*, *amylovorus*, *casei*, *fermentum*, *johnsonii*, *plantarum*, *reuteri*, *salivarius*), *Leuconostoc* (*citreum*, *fallax*, *lactis*, *mesenteroides*), *Listeria monocytogenes*, *Microbacterium hominis*, *Neisseria canis*, *Olsenella* (*profusa*, *uli*), *Proteus* (*mirabilis*, *vulgaris*), *Pseudomonas aeruginosa*, *Putida vulgaris*, *Ralstonia solanacearum*, *Salmonella* (*Enteritidis*, *paratyphi*, *typhi*, *typhimurium*), *Serratia marcescens*, *Shigella* (*dysenteriae*, *sonnei*), *Staphylococcus* (*aureus*, *epidermidis*), *Streptococcus* (*aureus*, *epidermidis*, *bovis*, *gallolyticus*, *hyointestinalis*, *porcinus*, *pyogenes*, *salivarius*), *Veillonella ratti*, *Vibrio cholerae*, *Weissella* (*confusa*, *hellenica*), *Xanthomonas oryzae*	Simplified: Data transformed into general Species categories	*A. niger*, *A. terreus*, *Aspergillus*, *B. brevis*, *B. cereus*, *B. licheniformis*, *B. subtilis*, *C. albicans*, *C. tropicalis*, *E. aerogenes*, *E. coli*, *E. faecalis*, *Enterococcus*, *F. solani*, *Fusarium*, *Ganoderma*, *K. pneumoniae*, *L. monocytogenes*, *P. aeruginosa*, *P. mirabilis*, *P. multocida*, *P. putida*, *P. vulgaris*, *Penicillium*, *S. aureus*, *S. dysenteriae*, *S. epidermidis*, *S. marcescens*, *S. paratyphi*, *S. typhi*, *Salmonella*, *Scedosporium*, *Shigella*, *V. cholerae*, *X. oryzae*, *Xanthomonas*

## Data Availability

Data can be found in Supplementary Material S1.

## Supplementary Materials

The following are available online at https://www.mdpi.com/article/10.3390/nano11071774/s1, Supplementary Material S1: Dataset. Supplementary Material S2: Data analysis, Figure S1: Seaborn pairplot correlation analysis of input variables with the outcome, Figure S2: Batch/experimental effect in modeling training. Each experiment signifies one study. The x axis demonstrates the data points that each study provided. The y axis demonstrates the actual and predicted zone of inhibition values. Experiment with Data points > 10, Figure S3: Batch/experimental effect in modeling training. Each experiment signifies one study. The x axis demonstrates the data points that each study provided. The y axis demonstrates the actual and predicted zone of inhibition values. Experiment with Data points < 10.

## Abbreviations

NPs	Nanoparticles
NMs	Nanomaterials
AL	Artificial Intelligence
ML	Machine Learning
MO NPs	Metal Oxide nanoparticles
ZnO NPs	Zinc Oxide nanoparticles
Fe_2_O_3_ NPs	Iron Oxide nanoparticles
Ag NPs	Silver nanoparticles
ROS	Reactive Oxygen Species
ECP	ExtraCellular Polymers
OD	Optical Density
ZOI	Zone Of Inhibition
MIC	Minimum Inhibitory Concentration
MBC	Minimum bactericidal Concentration
CFU	Colony Forming Unit
RF	Random Forest
ENR	Elastic Net Regression
SVM	Supervised Vector Machine
LASSO	Least Absolute Shrinkage and Selection Operator
RR	Ridge Regression
