# Supplementary material for: A Machine Learning Tool to Predict the Antibacterial Capacity of Nanoparticles
Source: Nanomaterials (Basel). 2021 Jul 7;11(7):1774. doi: 10.3390/nano11071774 (PMC8308172; doi:10.3390/nano11071774)
Supplement: Supplementary file 1 [file nanomaterials-11-01774-s001.zip › nanomaterials-1248289-Supplementary Materials (S2)-Data analysis.pdf]

nanomaterials

Supplementary Materials

A Machine Learning Tool to Predict the Antibacterial Capacity of Nanoparticles

Mahsa Mirzaei [1], Irini Furxhi [1,2,*], Finbarr Murphy [1,2] and Martin Mullins [1]

1 Department of Accounting and Finance, Kemmy Business School, University of Limerick, V94PH93 Limerick, Ireland; mahsa.mirzaei@ul.ie (M.M.); finbarr.murphy@ul.ie (F.M.); martin.mullins@ul.ie (M.M.)
2 Transgero Limited, Cullinagh, Newcastle West, Co., V42V384 Limerick, Ireland
* Correspondence: irini.furxhi@ul.ie; Tel.: +353-85-106-9771

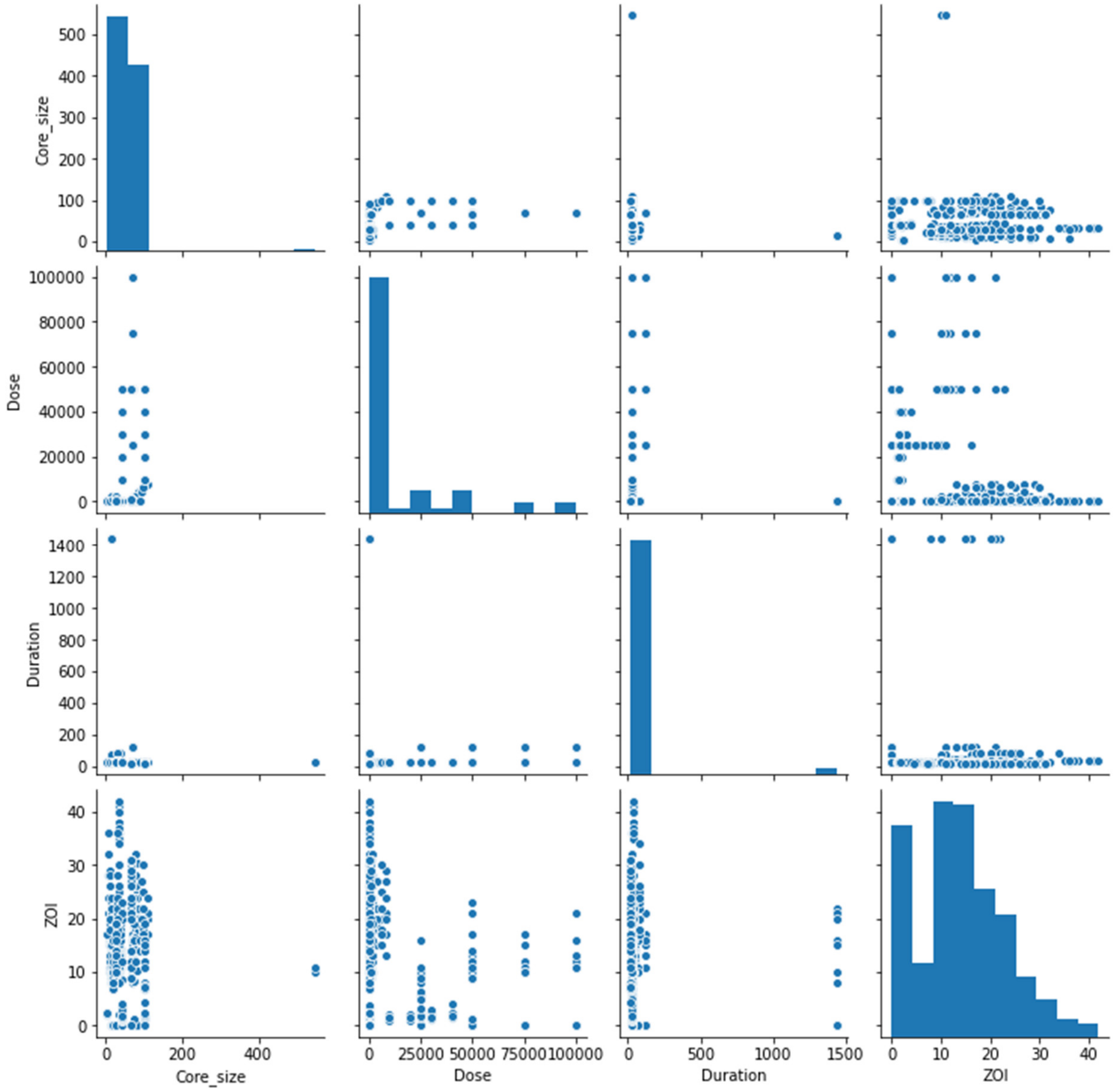

Figure S1. Seaborn pairplot correlation analysis of input variables with the outcome.

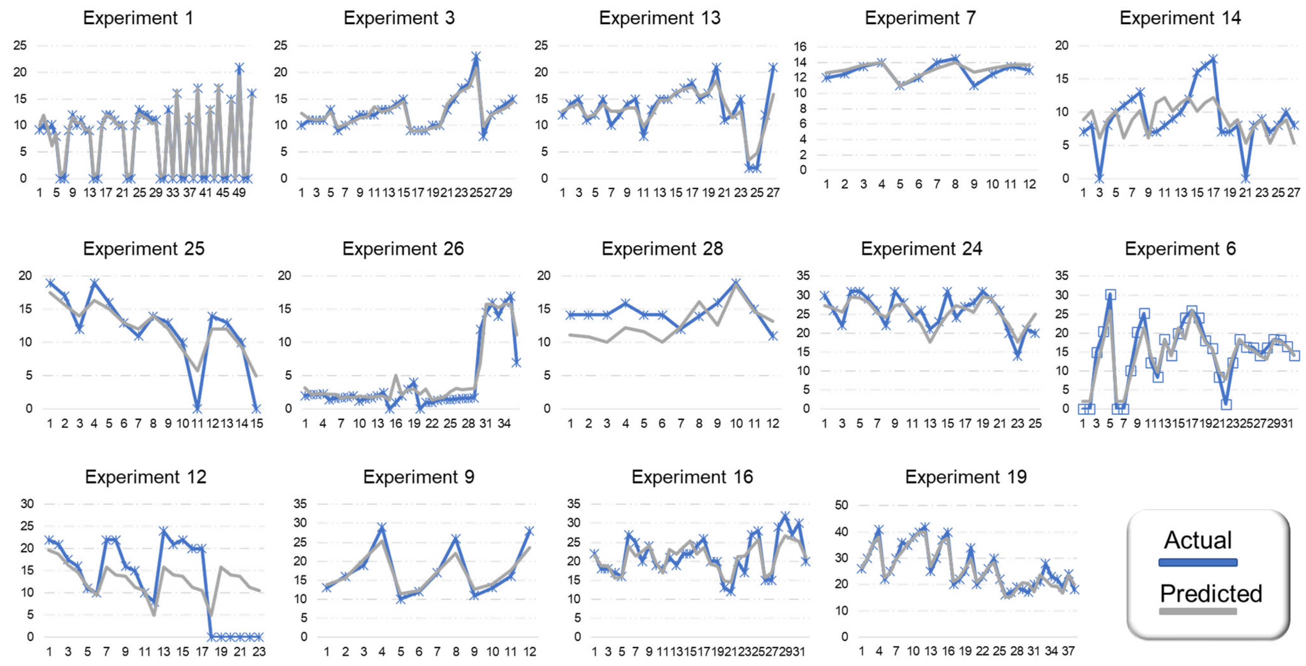

Figure S2. Batch/experimental effect in modelling training. Each experiment signifies one study. The x axis demonstrates the data points that each study provided. The y axis demonstrates the actual and predicted zone of inhibition values. Experiment with Data points >10.

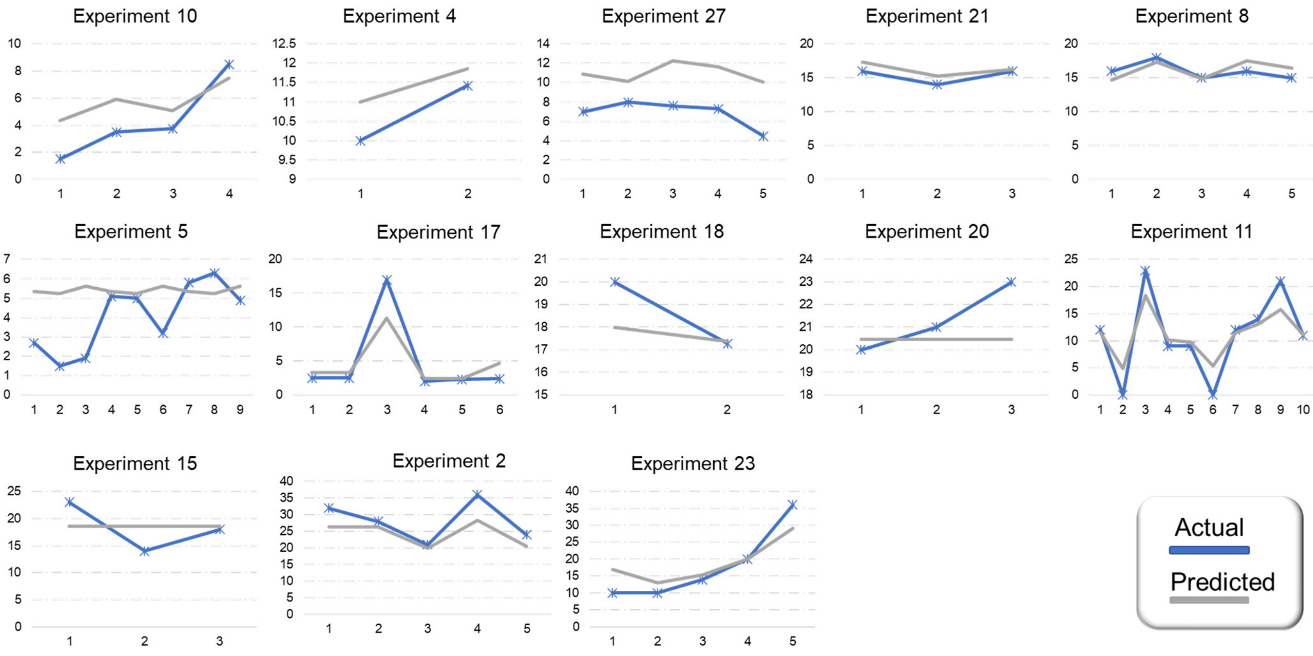

Figure S3. Batch/experimental effect in modelling training. Each experiment signifies one study. The x axis demonstrates the data points that each study provided. The y axis demonstrates the actual and predicted zone of inhibition values. Experiment with Data points <10.

It is evident from Figure S2 and Figure S3 that experiments (studies) that provided few instances (data points <10) are not captured well by the random forest model. However, this demonstartes a realistic scenario where instances are taken as in the case of integrated databases. We did not stratified the dataset artificially in order to demonstrate the importance of the data volume provided by individual studies. However, we could have include datapoints from each experiment into the training model to generalize it.
